# Dynamic association of human mRNP proteins with mitochondrial tRNAs in the cytosol

**DOI:** 10.1261/rna.066738.118

**Published:** 2018-12

**Authors:** Beáta E. Jády, Amandine Ketele, Tamás Kiss

**Affiliations:** 1 Laboratoire de Biologie Moléculaire Eucaryote du CNRS, Centre de Biologie Intégrative, Université de Toulouse, CNRS, UPS, 31062 Toulouse Cedex 9, France; 2 Biological Research Centre, Hungarian Academy of Sciences, Szeged, 6726 Hungary

**Keywords:** mRNA-binding protein, mRNP, cold shock domain protein, YBX1, mitochondrial tRNA

## Abstract

Cytoplasmic localization, stability, and translation of mRNAs are controlled by their dynamic association of numerous mRNA-binding (mRNP) proteins, including cold shock domain (CSD)-containing proteins, heterogeneous nuclear ribonucleoproteins (hnRNPs), and serine/arginine-rich (SR) proteins. Here, we demonstrate that the most abundant human mRNP protein, the CSD-containing Y-box-binding protein 1 (YBX1), the closely related YBX3 protein, and other mRNP proteins, such as SRSF1, SRSF2, SRSF3, hnRNP A1, and H, specifically and efficiently interact with overlapping sets of mitochondrial tRNAs (mt tRNAs). In vitro reconstitution and in vivo binding experiments show that YBX1 recognizes the D- and/or T-stem–loop regions of mt tRNAs through relying on the RNA-binding capacity of its CSD. Cell fractionation and in vivo RNA–protein cross-linking experiments demonstrate that YBX1 and YBX3 interact with mt tRNAs in the cytosol outside of mitochondria. Cell fractionation and fluorescence in situ hybridization experiments provide evidence that mitochondrial autophagy promotes the release of mt tRNAs from the mitochondria into the cytoplasm. Association of mRNP proteins with mt tRNAs is highly dynamic; it is rapidly increased upon transcription inhibition and decreased during apoptosis. Although the cytoplasmic function of mt tRNAs remains elusive, their dynamic interactions with key mRNA-binding proteins may influence cytoplasmic mRNA stability and/or translation.

## INTRODUCTION

Human Y-box-binding proteins YBX1, YBX3, and the germ cell–specific YBX2 belong to the superfamily of cold shock domain (CSD) proteins (Wolffe et al. 1992; Graumann and Marahiel 1998). CSD is an evolutionarily highly conserved, single-stranded nucleic acid–binding domain encompassing two RNA-recognition motifs, RNP-1 and RNP-2, which are common to many RNA-binding proteins (Kloks et al. 2002). Through interacting with nucleic acids, the Y-box-binding proteins function in a broad variety of cellular processes, including nuclear DNA replication, DNA repair, transcription, and mRNA processing (Eliseeva et al. 2011; Lyabin et al. 2014). Together with numerous hnRNP proteins and serine/arginine-rich (SR) proteins, YBX1 and YBX3 bind to the newly synthesized precursor mRNAs (pre-mRNAs). They regulate pre-mRNA splicing and polyadenylation, promote mRNA export to the cytoplasm, and control cytoplasmic mRNA translation, stability, and localization (Eliseeva et al. 2011; Lyabin et al. 2014; Singh et al. 2015). Besides polyadenylate-binding protein 1 (PABP1), YBX1 is the major interactor of cytoplasmic mRNAs (Minich et al. 1993; Evdokimova et al. 1995; Singh et al. 2015). Dynamic association of YBX1 with mRNAs controls the cytoplasmic stability and translation of a large set of mRNAs (Evdokimova et al. 1998, 2006; Lyabin et al. 2014). The regulatory mechanisms controlling association of YBX1 and other mRNP proteins with cytoplasmic mRNAs is poorly understood. Recently, cytoplasmic (cyt) tRNA fragments (tRFs) produced by stress-activated angiogenin have been found to possess high affinity to YBX1 and have been demonstrated to destabilize YBX1-associated mRNAs by competitively displacing them from YBX1 (Goodarzi et al. 2015).

Mitochondria are double membrane–bound cytoplasmic organelles that function primarily in cellular energy production though ATP synthesis by oxidative phosphorylation. The 16.6-kb human circular mitochondrial DNA encodes 13 mitochondrial proteins involved in oxidative phosphorylation, two mitochondrial ribosomal RNAs (12S and 16S rRNAs), and the complete set of 22 mt tRNAs sufficient for mitochondrial protein synthesis (Hällberg and Larsson 2014). The limited protein-coding capacity of mitochondrial DNA is compensated by selective importation of about 1500 nuclear-encoded proteins from the cytoplasm (Neupert and Herrmann 2007). All human mt tRNAs are synthesized within and processed from long polycistronic transcripts by RNase P and ELAC2 within the mitochondrion (Dubrovsky et al. 2004; Holzmann et al. 2008; Brzezniak et al. 2011; Rossmanith 2011). To produce functional mt tRNAs, several internal nucleotides are covalently modified and the tRNA nucleotidyltransferase adds the 3′-terminal CCA trinucleotide (Nagaike et al. 2001; Salinas-Giegé et al. 2015). The mature mt tRNAs possess noncanonical cloverleaf structures distinct from cyt tRNAs (Watanabe 2010; Suzuki et al. 2011).

The cellular function of mt tRNAs has long been confined to mitochondrial protein synthesis. Recent studies, however, raised the fascinating concept that mt tRNAs may have additional, unexpected regulatory functions outside of mitochondria. The Yang laboratory reported that human mt tRNAs function as negative regulators of apoptosis through binding to the mitochondrial apoptosome activator cytochrome *c* (Cyt *c*) (Mei et al. 2010). Docking of mt tRNAs prevents Cyt *c* binding to the cytosolic protease activating factor 1 (Apaf-1), which is essential for Apaf-1-mediated caspase activation and apoptosis (van Raam and Salvesen 2010). Cyt *c* and mt tRNAs are likely released from the mitochondria in response to intrinsic apoptotic signals that can promote mitochondrial membrane permeabilization and lesion formation (Zhan et al. 2013). Other studies suggested that human mt tRNAs, or at least some of them, are present in the cytosol even under normal physiological conditions. Human mt tRNA^Met^ has been found to specifically interact with argonaute-2 (Ago2), a key component of the cytoplasmic RNA-induced silencing complex (Maniataki and Mourelatos 2005). More recently, our laboratory demonstrated that the human polypyrimidine tract-binding mRNP protein PTB and its tissue-specific paralogs PTB2 and PTB3 bind with great specificity to mt tRNA^Thr^ in the cytoplasm (Marnef et al. 2016). PTB interaction with mt tRNA^Thr^ is augmented during apoptosis, pointing to a potential participation of the PTB/mt tRNA^Thr^ complex in apoptosis.

In this study, we demonstrate that a fraction of human HeLa mt tRNAs accumulate in the cytoplasm where they specifically interact with abundant mRNP proteins, including the YBX1 and YBX3 CSD proteins, the SRSF1, SRSF2, and SRSF3 SR proteins, and the hnRNP A1 and H proteins. Cytoplasmic association of mt tRNAs with mRNP proteins is highly dynamic, it is rapidly augmented in transcriptionally arrested cells, and is reduced in apoptotic cells. Although the function of cytoplasmic association of mt tRNAs and mRNP proteins remains unknown, our observations reinforce the currently emerging idea that besides supporting mitochondrial protein synthesis, mt tRNAs also possess other functions in the cytoplasm.

## RESULTS

### Human YBX1 and YBX3 interact with mt tRNAs

**FIGURE 1.**
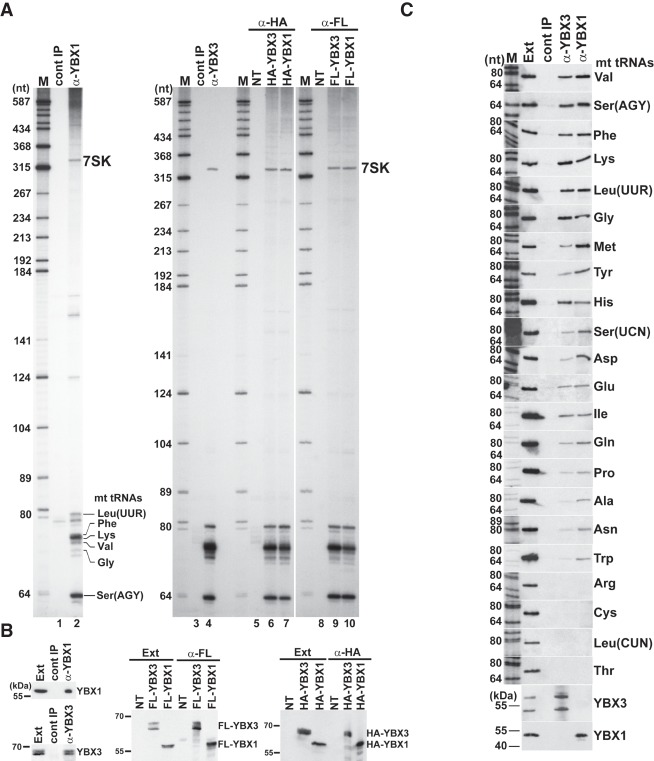
Human YBX1 and YBX3 interact with mt tRNAs. (*A*) Small RNAs associated with YBX1 and YBX3. HeLa cellular RNAs coimmunopurified with endogenous or transiently expressed HA- and Flag-tagged YBX1 and YBX3 were 3′ end-labeled and analyzed on 6% sequencing gels. Control IPs with nonimmune sera (cont IP) or from nontransfected cell extracts (NT) are shown. The 7SK snRNA and YBX1-associated mt tRNAs identified by direct chemical sequencing are indicated. (Lanes M) Size markers in nucleotides. (*B*) Western blot analyses. IP of HeLa endogenous and transiently expressed epitope-tagged YBX1 and YBX3 was confirmed by YBX1- and YBX3-specific antibodies. (Ext) HeLa cell extracts. Positions of marker proteins are indicated. (*C*) Copurification of HeLa mt tRNAs with YBX1 and YBX3 was measured by northern blot analyses with sequence-specific oligonucleotide probes. IP of YBX1 and YBX3 was confirmed by western blotting. (Ext) HeLa cell extract (50× dilution). (Lane *M*) Size markers.

The human CSD proteins YBX1 and YBX3 have been identified as potential interactors of the 7SK transcriptional regulatory snRNA (Hogg and Collins 2007; McNamara et al. 2016; B Jady, A Ketele, T Kiss, unpubl.). To confirm in vivo association of YBX1 and YBX3 with 7SK, both proteins were immunoprecipitated with specific antibodies from a HeLa total cell extract depleted of large RNPs. RNAs copurified with YBX1 and YBX3 were 3′ end-labeled with [5′-^32^P]pCp and T4 RNA ligase and size-fractionated on a 6% sequencing gel (Fig. 1A, lanes 2,4). Immunoprecipitation (IP) of both YBX1 and YBX3 pulled down the 7SK snRNA and, more relevant to this study, also recovered a group of 60- to 80-nt-long small RNAs. The same set of RNAs were recovered upon IP of transiently expressed HA- and Flag-tagged YBX1 and YBX3 with anti-HA and anti-Flag antibodies (lanes 6,7,9,10). Moreover, a similar RNA profile was obtained upon analysis of RNAs coimmunopurified with transiently expressed TAP-tagged YBX3 (Supplemental Fig. S1). IP of endogenous and transiently expressed epitope-tagged YBX1 and YBX3 proteins was confirmed by western blot analysis (Fig. 1B).

To determine the identity of small RNAs copurified with YBX1, several 3′ end-labeled RNAs were isolated from the gel and subjected to direct chemical sequencing. Gel electrophoretic fractionation of the base-specific degradation products provided clear sequencing patterns for six RNAs that, to our surprise, corresponded to mt tRNAs Phe, Lys, Ser(AGY), Val, Leu(UUR), and Gly (Supplemental Fig. S2A). In the sequencing ladders, the uridine reactions contained a few gaps that likely indicate hydrazine-resistant pseudouridines, suggesting that mt tRNAs associated with YBX1 are post-transcriptionally modified. Next, 3′ end race experiments followed by sequence analyses demonstrated that most YBX1-associated mt tRNAs Phe, Lys, and Gln carry the nontemplated 3′-terminal CCA ribonucleotides of mature tRNAs, although ∼30% of the obtained sequences lacked the last A, CA, CCA, or NCCA residues (Supplemental Fig. S2B, and data not shown). This indicates that the YBX1-associated mt tRNAs are correctly processed, but they undergo slight 3′ end trimming in the cell or in the extract used for IP.

**FIGURE 2.**
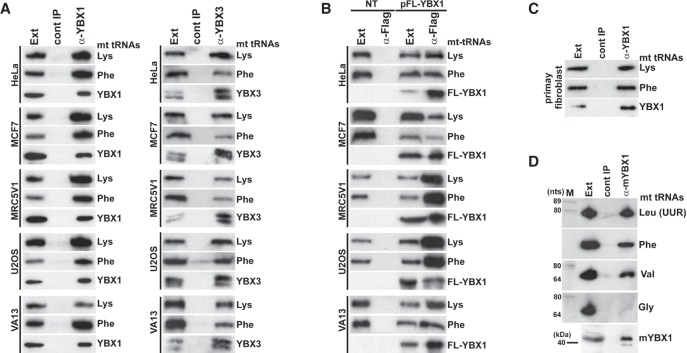
Conservation of YBX1 and YBX3 interaction with mt tRNAs in human and mouse cells. (*A*) YBX1 and YBX3 interaction with mt tRNAs in human cell lines. YBX1 and YBX3 were immunoprecipitated from extracts (Ext) of HeLa, MCF7, MRC5V1, U2OS, and VA13 cells. IP of YBX1 and YBX3 and copurification of mt tRNA^Lys^ and mt tRNA^Phe^ were monitored by western and northern blot analyses, respectively. (*B*) Transiently expressed Flag-tagged YBX1 interacts with endogenous mt tRNA^Lys^ and mt tRNA^Phe^ in human HeLa, MCF7, MRC5V1, U2OS, and VA13 cells. FL-YBX1 was immunoprecipitated with an anti-Flag antibody from extracts (Ext) of nontransfected (NT) and transfected (pFL-YBX1) cells. IP of FL-YBX1 and co-IP of mt tRNA^Lys^ and mt tRNA^Phe^ were monitored by western and northern blotting. (*C*) YBX1 interacts with mt tRNA^Lys^ and mt tRNA^Phe^ in human primary fibroblast cells. Please note that fibroblast cells do not express YBX3. (*D*) Mouse YBX1 binds to mt tRNAs. Mouse YBX1 (mYBX1) was immunoprecipitated from an L929 cell extract with a specific antibody, and coprecipitation of four selected mt tRNAs was tested.

Northern blot analysis was used to compare the interactions of mt tRNAs with YBX1 and YBX3 (Fig. 1C). Apart from mt tRNAs Arg, Cys, Leu(CUN), and Thr, all mt tRNAs copurified with YBX1 and YBX3, albeit with different efficiencies. In contrast, we failed to detect any of the tested cyt tRNAs Asp, Leu, Glu, Lys, Phe, and the Y5 small cytoplasmic RNA among YBX1- and YBX3-associated RNAs (Supplemental Fig. S3). IP of endogenous YBX1, YBX3, and transiently expressed FL-YBX1 from human MCF7, MRC5V1, U2OS, VA13, and primary fibroblast cell extracts recovered mt tRNAs Lys and Phe (Fig. 2A–C). Likewise, IP of mouse YBX1 from a L929 cell extract recovered three out of the four tested mt tRNAs (Fig. 2D). We concluded that association of human mt tRNAs with YBX1 and YBX3 is conserved at least in human and mouse cells.

### YBX1 and YBX3 directly interact with mt tRNAs

**FIGURE 3.**
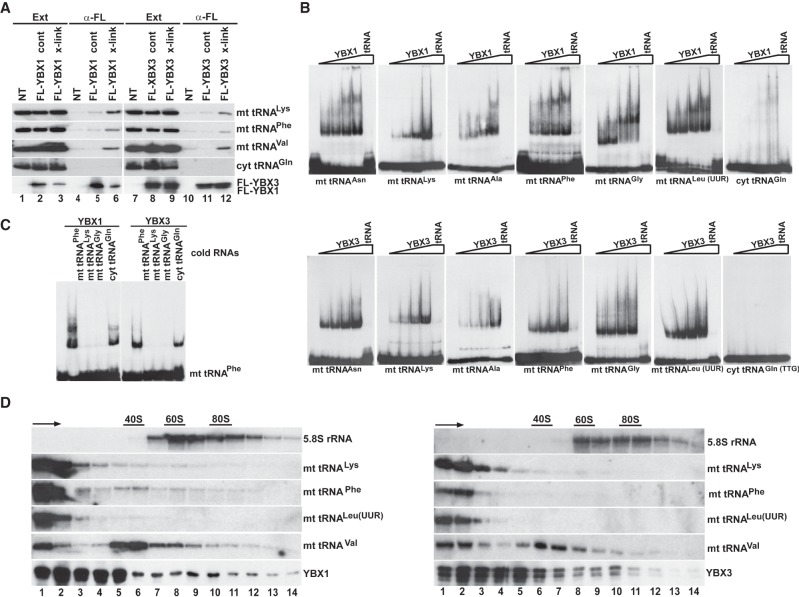
YBX1 and YBX3 directly interact with mt tRNAs. (*A*) In vivo RNA–protein cross-linking. Extracts (Ext) were prepared from formaldehyde-treated (x-link) or nontreated (cont) HeLa cells expressing FL-YBX1 or FL-YBX3. After IP with an anti-Flag antibody under stringent conditions, recovery of FL-YBX1 and FL-YBX3 was monitored by western blotting, and co-IP of mt tRNAs Lys, Phe, and Val, as well as cyt tRNA^Gln^, was measured by northern blotting. (NT) Nontransfected cells. (*B*) Gel shift analysis. In vitro synthesized ^32^P-labeled mt and cyt tRNAs (1 fmol) were incubated without or with increasing amounts of recombinant YBX1 and YBX3 proteins (0.1, 0.2, 0.8, and 4 pmol) in the absence or presence of 50 fmol of cold competitor tRNA (tRNA). The resulting complexes were analyzed on 5% native acrylamide gels. (*C*) Cross-competition of YBX1 and YBX3 binding to mt tRNA^Phe^ by other mt tRNAs. Labeled mt tRNA^Phe^ (1 fmol) was incubated without or with 4 pmol of recombinant YBX1 and YBX3 in the absence or presence of cold competitor mt tRNAs (50 fmol) as indicated. (*D*) Sedimentation analyses of YBX1- and YBX3-mt RNA complexes. HeLa cell extracts were fractionated by utracentrifugation on 10%–50% sucrose gradients. Each gradient was divided into 14 fractions before IP of YBX1 and YBX3. Co-IP of selected mt tRNAs was monitored by northern blotting. Distribution of 5.8S rRNA is shown. Positions of ribosomal particles were determined by UV absorption profiling. Arrows indicate sedimentation directions.

To demonstrate that YBX1 and YBX3 interact with mt tRNAs in living cells, we performed in vivo RNA–protein cross-linking experiments (Fig. 3A). Because the available YBX1 and YBX3 antibodies performed poorly under stringent IP conditions required to destroy noncovalent RNA–protein interactions, transiently expressed Flag-tagged YBX1 and YBX3 were immunoprecipitated with an anti-Flag antibody. Before extract preparation, the transfected cells were treated with formaldehyde to fix in vivo RNA–protein interactions (Niranjanakumari et al. 2002). Without formaldehyde cross-linking, IP of FL-YBX1 and FL-YBX3 under harsh conditions failed to recover significant amounts of mt tRNAs Lys, Phe, and Val (lanes 5,11). In contrast, in vivo cross-linking significantly increased copurification of the tested mt tRNAs with both YBX1 and YBX3 (lanes 6,12), confirming that YBX1 and YBX3 directly interact with mt tRNAs in living cells. As expected, neither YBX1 nor YBX3 showed detectable association with cyt tRNA^Gln(UUG)^ even after formaldehyde cross-linking.

To confirm direct interaction of YBX1 and YBX3 with mt tRNAs, we performed gel electrophoretic mobility shift assays (Fig. 3B). In vitro synthesized internally labeled mt tRNAs Asn, Lys, Ala, Phe, Gly, and Leu(UUR) were incubated with increasing amounts of recombinant YBX1 and YBX3 proteins, and the resulting complexes were analyzed by native gel electrophoresis. Each mt tRNA showed YBX1 and YBX3 concentration-dependent mobility retardation that was abolished by inclusion of the corresponding cold competitor mt tRNA. The supershifts frequently observed in YBX1 reactions likely reflect in vitro YBX1 dimerization (Evdokimova et al. 1995). In contrast to mt tRNAs, the cyt tRNA^Gln(TTG)^ failed to interact with YBX1 and YBX3. In vitro association of mt tRNA^Phe^ with YBX1 and YBX3 was disrupted by inclusion of excessive amounts of other cold mt tRNAs, such as Phe, Lys, and Gly, but remained unaffected in the presence of cold cyt tRNA^Gln(TTG)^ (Fig. 3C). These results demonstrate that YBX1 and YBX3 form direct and specific interactions with mt tRNAs.

Next, HeLa cell extracts were fractionated by sucrose gradient velocity sedimentation to learn about the natures of YBX1- and YBX3-mt tRNA complexes (Fig. 3D). The gradients were divided into 14 fractions and upon IP of YBX1 and YBX3 from each fraction (bottom panels), copurification of mt tRNAs Lys, Phe, Leu(UUR), and Val were monitored by northern blot analyses (middle panels). YBX1 and YBX3 were found mostly in the upper parts of the gradients (5S–40S), but consistent with their proposed role in mRNA translation regulation, they were also detected in the lower part of the gradient containing rapidly sedimenting large complexes. The mt tRNAs Lys, Phe, and Leu(UUR) coprecipitated with YBX1 and YBX3 predominantly from the top two fractions, suggesting that they form simple heterodimeric complexes with YBX1 and YBX3. Supporting this notion, mass spectrometry failed to identify additional proteins coprecipitated with YBX1- and YBX3-mt tRNA complexes. Interestingly, only a fraction of mt tRNA^Val^ was detected in low mobility YBX1 and YBX3 complexes, more than half of mt tRNA^Val^ associated with YBX1 and YBX3 was incorporated into large 35S–50S complexes of unknown nature. Recently, mt tRNA^Val^ has been identified as an integral component of the 39S large subunit of human mitochondrial ribosomes (Brown et al. 2014). However, YBX1 and YBX3 are absent from mitochondria (see below), excluding their association with mitochondrial ribosomes.

### RNA and protein elements supporting YBX1 interactions with mt tRNAs

**FIGURE 4.**
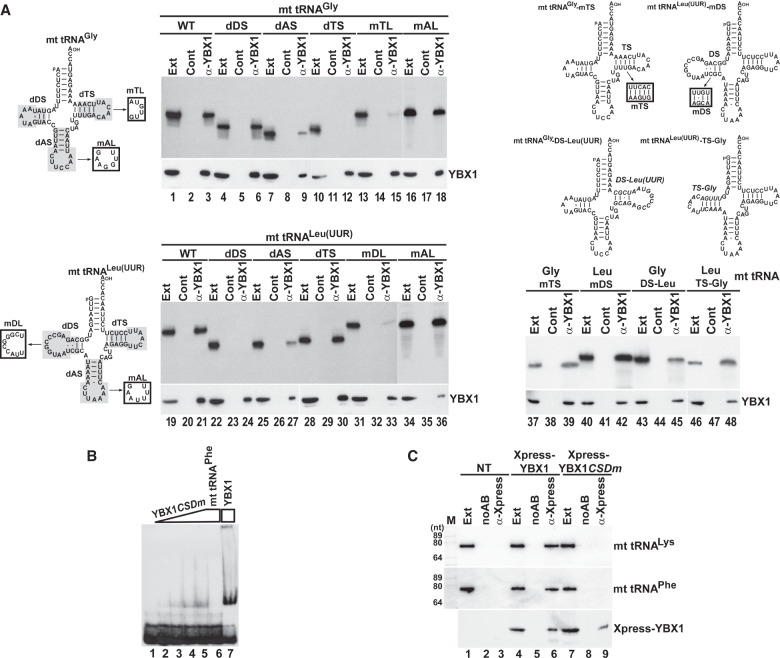
Protein and RNA elements directing YBX1 association with mt tRNA^Gly^ and mt tRNA^Leu(UUR)^. (*A*) YBX1 binding is directed by the D and T stem–loop of mt tRNA^Leu(UUR)^ and mt tRNA^Gly^, respectively. About 2 fmol of internally labeled wild-type and mutant mt tRNAs carrying internal truncations (shaded), sequence alterations (boxed) or other mt tRNA elements (italics) were mixed with 2 µg of cold *E. coli* tRNA and transfected into HeLa cells. After 24 h of incubation, endogenous YBX1 was immunoprecipitated and co-IP of labeled tRNAs was tested. (*B*) Gel shift analyses. In vitro transcribed labeled mt tRNA^Phe^ (1 pmol) was incubated without or with 0.2, 0.4, 1.5, and 4 pmol of recombinant mutant (YBX1*CSDm*) and 4 pmol of wild-type YBX1 in the absence or presence of cold mt tRNA^Phe^ (50 pmol) before analysis on a 5% native gel. (*C*) Intact CSD is required for in vivo association of YBX1 with mt tRNA^Lys^ and mt tRNA^Phe^. Transiently expressed Xpress-YBX1 and Xpress-YBX1*CSDm* were immunoprecipitated from transfected or nontransfected (NT) cell extracts and analyzed by western blotting. Co-IP of mt tRNAs Lys and Phe was monitored by northern blotting.

To determine the elements of mt tRNAs directing YBX1 binding, in vitro synthesized internally labeled wild-type and mutant mt tRNAs Gly and Leu(UUR) were transfected into HeLa cells (Fig. 4A). After 24 h of incubation, endogenous YBX1 was immunoprecipitated and copurification of the labeled test RNAs was monitored by gel electrophoresis followed by autoradiography. IP of YBX1 was confirmed by western blot analysis. All transfected RNAs proved to be stable in HeLa cells (lanes Ext). As expected, both wild-type tRNAs interacted with YBX1 (lanes 3,21). Removal of the D stem–loop (dDS) had no effect on YBX1 binding to the truncated mt tRNA^Gly^ (lane 6), but fully abolished YBX1 interaction with the shortened mt tRNA^Leu(UUR)^ (lane 24). Elimination of the T stem–loop (dTS) interrupted YBX1 binding to mt tRNA^Gly^ (lane 12), but failed to influence YBX1 interaction with mt tRNA^Leu(UUR)^ (lane 30). In line with these observations, alteration of the T and D loop sequences (mTL and mDL) of mt tRNA^Gly^ and mt tRNA^Leu(UUR)^, respectively, abolished association of the mutant mt tRNAs with YBX1 (lanes 15,33). Deletion of the anticodon (A) stem–loop (dAS) inhibited interaction of both truncated mt tRNAs with YBX1 (lanes 9,27). However, computer-mediated structure predictions indicated that in contrast to removal of the T and D stem–loops, deletion of the A stem–loops interferes with proper folding of the truncated mt tRNAs (Supplemental Fig. S4). Alteration of the anticodon loop sequences (mAL) had no influence on the YBX1 binding capacities of the mutant mt tRNA^Gly^ and mt tRNA^Leu(UUR)^ (lanes 18,36), suggesting that besides promoting correct RNA folding, the A stem–loop has no or little impact on YBX1 binding. Thus, we concluded that YBX1 binding is directed primarily by the D stem–loop region of mt tRNA^Leu(UUR)^ and the T stem–loop of mt tRNA^Gly^. Nucleotide alterations that maintained the double-stranded nature of the D stem of tRNA Leu(UUR) (mDS) or the T stem of tRNA Gly (mTS) had no effect on the YBX1-binding capacities of the mutant RNAs (lanes 39,42). Swapping the T and D stem–loops of tRNAs Gly and Leu(UUR) for the Leu(UUR) D and the Gly T stem–loops, respectively, maintained in vivo YBX1 binding, further demonstrating that these elements contain the key structural information for YBX1 recognition (lanes 45,48). It is noteworthy that PTB binding to mt tRNA^Thr^ is also coordinated by the D and T stem–loops of the tRNA (Marnef et al. 2016).

To test the importance of the CSD of YBX1 in mt tRNA binding, we performed gel-shift assays (Fig. 4B). In vitro synthesized labeled mt tRNA^Phe^ was incubated with a mutant recombinant YBX1 protein (YBX1*CSDm*) carrying the W65G, F66A, F74A, F85A, and H87G CSD point mutations that are predicted to interfere with RNA ligand binding (Max et al. 2007; Sachs et al. 2012). Compared to wild-type YBX1 (lane 7), the mutant YBX1*CSDm* showed weak in vitro association with mt tRNA^Phe^ (lanes 2–5). We also assayed the in vivo mt tRNA binding capacity of the mutant YBX1*CSDm* protein expressed in HeLa cells (Fig. 4C). In contrast to transiently expressed Xpress-tagged wild-type YBX1 (lane 6), the mutant Xpress-YBX1*CSDm* failed to recover HeLa mt tRNAs Lys and Phe (lane 9) upon IP, demonstrating that mt tRNA binding is coordinated by the CSD of YBX1.

### YBX1 and YBX3 interact with mt tRNAs in the cytosol outside of mitochondria

**FIGURE 5.**
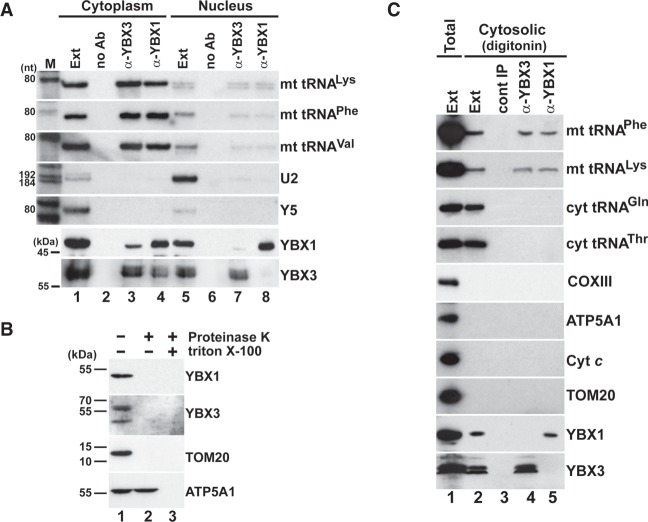
YBX1 and YBX3 associate with mt tRNAs in the cytosol. (*A*) Cell fractionation. YBX1 and YBX3 were immunoprecipitated from HeLa cytoplasmic and nuclear soluble extracts (*lower* panels). Co-IP of mt tRNAs was monitored by northern blot analyses (*upper* panels). (Lanes no Ab) Control IPs without antibody. Distribution of the U2 nuclear and the Y5 cytoplasmic RNAs was also tested. (*B*) YBX1 and YBX3 are not detectable in HeLa mitochondria. Crude mitochondrial fraction (lane *1*) was digested with proteinase K in the absence (lane *2*) and presence (lane *3*) of Triton X-100 before western blot analyses of YBX1, YBX3, and the mitochondrial marker proteins TOM20 and ATP5A1. (*C*) HeLa YBX1- and YBX3-mt tRNA complexes are present in a cytosolic extract devoid of mitochondrial contamination. A HeLa cytosolic extract was prepared by gentle digitonin extraction (lane *2*) and its protein (YBX1, YBX3, ATP5A1, Cyt *c*, and TOM20), mRNA (COXIII), and tRNA (mt tRNA^Phe^, mt tRNA^Lys^, cyt tRNA^Gly^ and cyt tRNA^Thr^) contents were compared to those of a diluted (10 times) total cell sonic extract (lane *1*). Co-IP of mt tRNAs Phe and Lys with YBX3 and YBX1 from the digitonin extract was confirmed.

After demonstration that YBX1 and YBX3 specifically and directly interact with mt tRNAs, we wanted to determine the subcellular localization of YBX1- and YBX3-mt tRNA complexes. Because human YBX1 and YBX3 accumulate in both the cytoplasm and the nucleoplasm, HeLa cells were first fractionated into crude cytoplasmic and nuclear fractions. To generate soluble cytoplasmic and nuclear extracts, mitochondria were depleted from the cytoplasmic fraction, and the nuclei were sonicated and clarified by centrifugation. Correctness of the cell fractionation was confirmed by monitoring the distributions of the Y5 cytoplasmic and the U2 nucleoplasmic RNAs (Fig. 5A, lanes 1,5). As expected, YBX1 and YBX3 were detected in both extracts, but their IP recovered mt tRNAs Lys, Phe, and Val predominantly from the cytosplasmic extract, suggesting that YBX1 and YBX3 interact with mt tRNAs in the cytosol (lanes 3,4). Significance of the observed weak interaction of YBX1 and YBX3 with mt tRNAs in the nuclear extract remains unclear (lanes 7,8). Co-IP of YBX1 and YBX3 from the cytoplasmic fraction could be explained by their copurification in mRNP particles.

YBX1 and YBX3 lack recognizable mitochondrial targeting sequences (Fukasawa et al. 2015; B Jady, A Ketele, T Kiss, unpubl.), and previous comprehensive analyses failed to detect these proteins within mitochondria (Mootha et al. 2003; Pagliarini et al. 2008; Gaston et al. 2009). Nevertheless, we assayed whether YBX1 and YBX3 are present in HeLa mitochondria (Fig. 5B). Similar to the mitochondrial marker proteins TOM20 and ATP5A1, both YBX1 and YBX3 were detectable in a crude mitochondrial preparation purified by sedimentation through a sucrose cushion (lane 1). However, proteinase K treatment of the mitochondrial fraction fully removed YBX1 and YBX3 as well as TOM20 that is located at the outer surface of the mitochondria (lane 2) (Chen et al. 2012). In contrast, ATP5A1 that resides in the mitochondrial inner membrane remained resistant to proteolysis in the absence of detergent. These results confirmed that YBX1 and YBX3 are missing from the mitochondria and further support the conclusion that they interact with mt tRNAs in the cytosol.

Next, we prepared a cytoplasmic soluble extract devoid of mitochondrial contaminations (Baghirova et al. 2015). To this end, HeLa cells were incubated with a digitonin-based extraction buffer that permeabilizes the plasma membrane but leaves intact the mitochondrial membranes (Fig. 5C). The resulting cytosolic extract contained both cytoplasmic (Gln and Thr) and mitochondrial (Phe and Lys) tRNAs as well as YBX1 and YBX3 (lane 2), but in contrast to the total sonic extract (lane 1), it lacked the ATP5A1, TOM20, and Cyt *c* mitochondrial marker proteins as well as the COXIII mitochondrial mRNA. IP of YBX1 and YBX3 from the digitonin extract recovered both mt tRNA^Phe^ and mt tRNA^Lys^, but failed to pull down cyt tRNAs Gln and Thr (lanes 4,5), further demonstrating that YBX1 and YBX3 interact with mt tRNAs in the cytosol outside of mitochondria. Comparing the relative mt tRNA levels in our digitonin and total sonic extracts indicated that ∼0.1% to 1% of cellular mt RNAs reside in the cytosol (Supplemental Fig. S5).

### Autophagic turnover of mitochondria supports mt tRNA release into the cytosol

**FIGURE 6.**
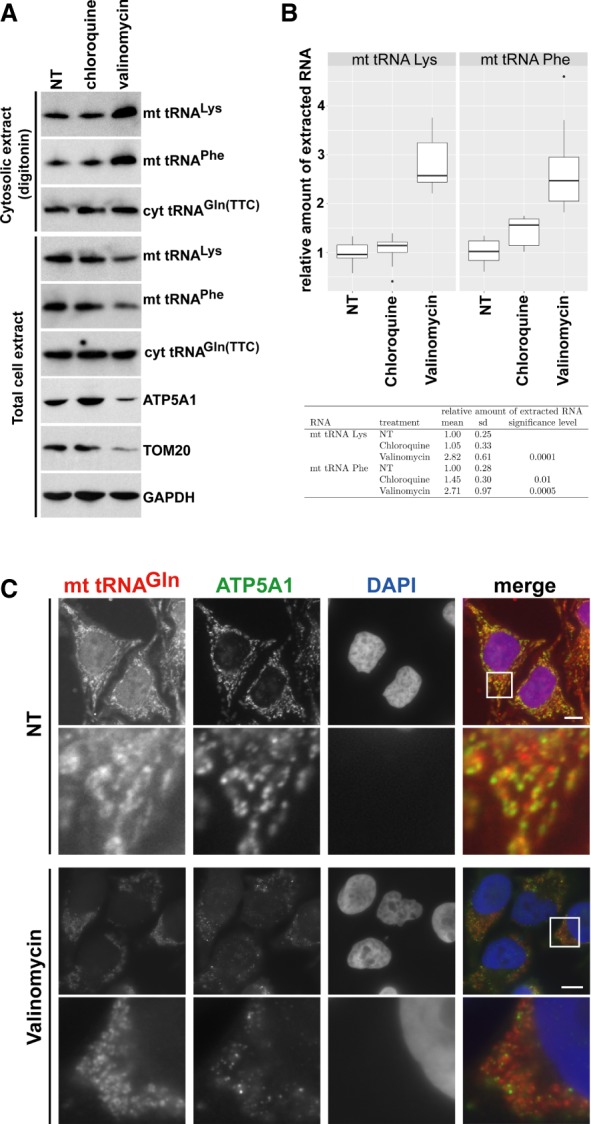
Induction of autophagy increases soluble cytosolic mt tRNA levels. (*A*) Accumulation of mt tRNAs in the cytosol of HeLa cells undergoing autophagy. Cytosolic and total cellular extracts were prepared from HeLa cells nontreated (NT) or treated with chloroquine and valinomycin by mild digitonin extraction or sonication, respectively. The levels of mt tRNAs Lys and Phe and the mitochondrial proteins ATP5A1 and TOM20 were measured by northern and western blotting. (*B*) Accumulation of mt tRNAs in the cytosolic extracts of HeLa cells either nontreated (NT) or treated with chloroquine or valinomycin was measured by slot blot analysis followed by PhosphorImager quantitation in eight independent experiments. Relative mt tRNA accumulations normalized to the levels of cyt tRNA^Gln^(TTC) are depicted by boxplots generated with the ggplot2 package (v2.2.1) in RStudio (v0.99.903 with R v3.3.1). The means and standard deviations (sd) are indicated. The obtained values for chloroquine- and valinomycin-treated cells were compared to the values obtained for NT control cells in a statistical analysis using Student's *t*-test. (*C*) Cytoplasmic distribution of mt tRNA^Gln^ after induction of mitophagy. HeLa cells, either treated or nontreated (NT) with valinomycin, were hybridized with a fluorescently labeled oligodeoxynucleotide specific for mt tRNA^Gln^. Mitochondria were stained with antibodies against ATP5A1. Bars, 10 µm. *Lower* panels show enhanced magnifications of the boxed cytoplasmic areas (13.4 × 13.4 µm). Nuclei were visualized with DAPI staining.

The observations that mt tRNAs interact with cytosolic RNA-binding proteins, such as YBX1 and YBX3 (this study), PTB, and Argonaut 2 (Maniataki and Mourelatos 2005; Marnef et al. 2016) raise the question how mt tRNAs can accumulate in the cytoplasm, because the mitochondrial membrane is impermeable for macromolecules. Dysfunctional or superfluous mitochondria are eliminated by selective lysosomal degradation, called mitochondrial autophagy or mitophagy (Kim et al. 2007; Ashrafi and Schwarz 2013; Rodger et al. 2018). To test whether mt tRNAs could be released into the cytosol during autophagic turnover of mitochondria, HeLa cells were treated with valinomycin that induces mitochondrial autophagy or with chloroquine that inhibits lysosomal activity (Georgakopoulos et al. 2017). Total cellular and cytosolic extracts were prepared from nontreated, valinomycin-treated, and chloroquine-treated cells by sonication and gentle digitonin extraction, respectively. In each extract, the concentrations of mt tRNAs Lys and Phe and mitochondrial marker proteins ATP5A1 and TOM20 were monitored (Fig. 6A). Confirming efficient induction of mitophagy, ATP5A1, TOM20, mt tRNA^Lys^, and mt tRNA^Phe^ levels were reduced in the valinomycin-treated total cell extract as compared to the nontreated and chloroquine-treated control extracts. In contrast, induction of mitophagy had no influence on the global cellular accumulation of GAPDH and cyt tRNA^Gln(TTC)^. Consistent with expectations, ATP5A1, TOM20, and the COXIII mRNA were missing from the digitonin cytosolic extract (see Supplemental Fig. S6). Accumulation of both mt tRNA^Lys^ and mt tRNA^Phe^ was increased in the cytosol of valinomycin-treated cells, contrary to their reduced global accumulation in these cells. Again, the cyt tRNA^Gln(TTC)^ levels remained unaltered in the cytosolic fractions of control and valinomycin-treated cells. Quantitation of eight independent experiments performed by slot blot analyses confirmed that the cytosolic concentrations of mt tRNAs Lys and Phe increased about 2.5-fold to threefold in HeLa cells undergoing valinomycin-induced mitophagy (Fig. 6B). Because the cytoplasm of valinomycin-treated cells lacked mitochondrial marker proteins and the COXIII mitochondrial mRNA, we excluded the possibility that elevated cytosolic mt tRNA accumulation was due to increased mitochondrial membrane fragility (Supplemental Fig. S6).

To further demonstrate that mitophagy promotes cytosolic accumulation of mt tRNAs, we compared the cytoplasmic distributions of mt tRNA^Gln^ in nontreated (NT) and valinomycin-treated HeLa cells by using fluorescence in situ hybridization microscopy (Fig. 6C). In control cells, the mt tRNA^Gln^ hybridization signals followed the overall patterns of mitochondrial structures highlighted by immunostaining with an anti-ATP5A1 antibody. The tRNA signals not overlapping with anti-ATP5A1 staining might indicate the cytoplasmic fraction of mt tRNA^Gln^. As expected, valinomycin treatment largely reduced the global accumulation of ATP5A1, and the remaining ATP5A1 showed less colocalization with mt tRNA^Gln^. The nonoverlapping fraction of mt tRNA^Gln^ had an uneven speckled distribution mainly around the remaining cytoplasmic mitochondria. Identical results were obtained upon visualization of the intracellular localization of mt tRNA^Phe^ in valinomycin-treated cells (Supplemental Fig. S7), supporting the idea that autophagic degradation of mitochondria promotes the release of mt tRNAs into the surrounding cytoplasm.

### Transcription inhibition increases YBX1 and YBX3 association with mt tRNAs

**FIGURE 7.**
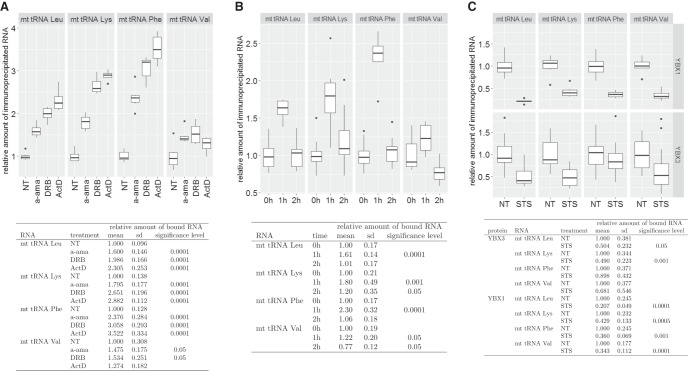
Dynamic association of YBX1 with mt tRNAs. (*A*) Transcription inhibition increases YBX1 association with mt tRNAs. Human HeLa cells were nontreated (NT) or treated with α-amanitin (a-ama), 5,6-dichloro-1-β-D-ribofuranosylbenzimidazole (DRB), or actinomycin D (ActD). After extract preparation, YBX1 was immunoprecipitated and coprecipitation of mt tRNAs Leu(UUR), Lys, Phe, and Val was measured by slot blot analyses and PhosphorImager quantification. For each transcription inhibitor, 12 independent inhibition, IP, and slot blot experiments were performed and the obtained relative hybridization intensity values were normalized to the means of values obtained for nontreated control cells. The means and standard deviations (sd) for each condition are indicated. The obtained values of treated cells were compared to the values obtained for NT control cells in a statistical analysis using Student's *t*-test. For conditions statistically different from the corresponding control condition, the significance levels are indicated. (*B*) DRB treatment of HeLa cells increases YBX1 association with mt tRNAs in a reversible manner. YBX1 association with mt tRNAs Leu(UUR), Lys, Phe, and Val was measured in nontreated HeLa cells (0 h), in cells grown for 1 h in the presence (1 h) and for another hour in the absence of DRB (2 h). Other details are identical to panel *A*. (*C*) YBX1 and YBX3 association with mt tRNAs is decreased in apoptotic cells. YBX3 and YBX1 were immunoprecipitated from extracts prepared from HeLa cells nontreated (NT) or treated with staurosporine (STS) for 4 h. Co-IP of mt tRNAs was measured as described in panel *A*.

We tested whether YBX1 and YBX3 form metabolically stable complexes with mt tRNAs or their interactions depend on the actual transcriptional or physiological conditions of the cell. HeLa cells were treated with α-amanitin (a-ama), 5,6-Dichloro-1-β-D-ribofuranosylbenzimidazole (DRB), and actinomycin D (ActD) to arrest nuclear RNA synthesis (Bensaude 2011). After extract preparation, YBX1 was immunoprecipitated and its association with mt tRNAs Lys, Phe, Leu(UUR), and Val was measured by slot blot analysis followed by PhosphorImager quantitation. For each interaction, we performed 12 independent transcription inhibition and IP experiments. After incubation with a-ama for 3 h or with DRB and ActD for 1 h, YBX1 showed increased associations with mt tRNAs Lys, Phe, and Leu(UUR) (Fig. 7A). Interestingly, mt tRNA^Val^ association with YBX1 only moderately increased in response to transcription inhibition, further supporting the assumption that mt tRNA^Val^ has a unique cytoplasmic function (see Fig. 3C).

Because the transcriptional inhibitory effect of DRB is reversible (Bensaude 2011), HeLa cells were grown for 1 h in the presence and an additional hour in the absence of DRB (Fig. 7B). One hour after DRB removal (2 h), the increased YBX1 and mt tRNA association levels (1 h) returned to the original or in the case of mt tRNA^Val^, even below the original level (0 h). We have previously observed that interaction of PTB with mt tRNA^Thr^ increases in apoptotic HeLa cells (Marnef et al. 2016). However, upon induction of apoptosis by STS administration, association of HeLa YBX1 and YBX3 with mt tRNAs was significantly reduced (Fig. 7C). Nevertheless, these results collectively demonstrate that YBX1 and YBX3 associate with mt tRNAs in dynamic and regulated manners.

### Interaction of mt tRNAs with various mRNP proteins

**FIGURE 8.**
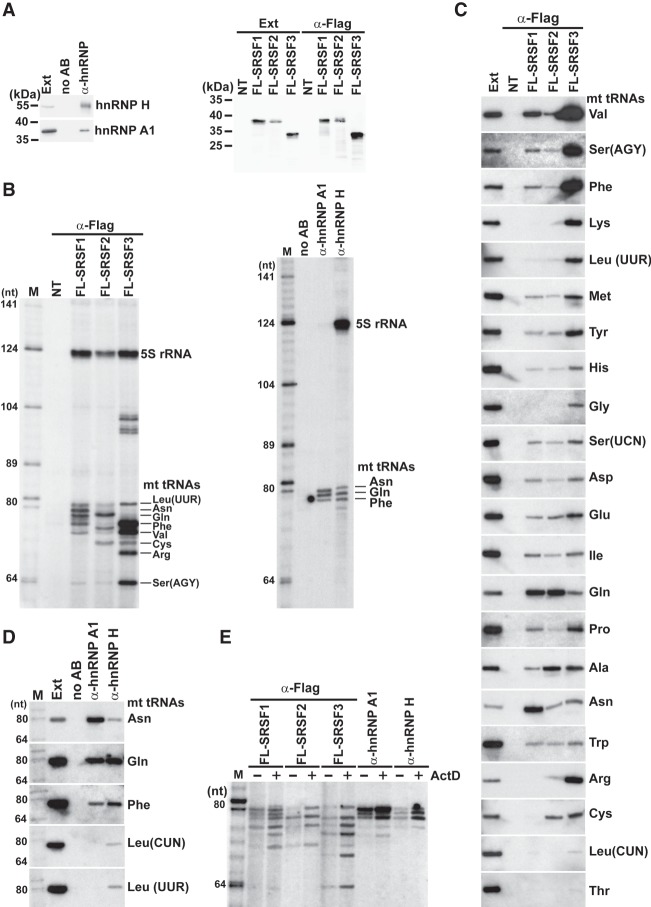
Interaction of mRNP proteins with HeLa mt tRNAs. (*A*) Western blot analyses. HeLa endogenous hnRNP A1 and H and transiently expressed Flag-tagged SRSF1, SRSF2, and SRSF3 proteins were immunoprecipitated and analyzed by western blotting. Control IPs from nontransfected cells (NT) or without antibody (no AB) are shown. (*B*) Small RNAs coimmunoprecipitated with Flag-tagged SRSF1, SRSF2, and SRSF3 and hnRNP A1 and H proteins were 3′ end-labeled and analyzed on 6% sequencing gels. (*C*) SR proteins interact with mt tRNAs. Co-IP of mt tRNAs with Flag-tagged SRSF1, SRSF2, and SRSF3 proteins was monitored by northern blot analyses. (Ext) Input extract (50× dilution). (*D*) Interaction of HeLa hnRNP A1 and H with mt RNAs. (*E*) Dynamic association of mRNP proteins with mt tRNAs. HeLa cells nontransfected (hnRNP A1 and H) or transfected with the pFL-SRSF1, pFL-SRSF2, or pFL-SRSF3 expression plasmids were divided into two fractions which were treated or nontreated with ActD. After extract preparation, the transiently expressed Flag-tagged SRSF1, SRSF2, and SRSF3 and endogenous hnRNP A1 and H proteins were immunoprecipitated and the copurified mt tRNAs were analyzed on a sequencing gel.

Our findings that human mt tRNAs specifically bind to human mRNP proteins YBX1, YBX3, and PTB prompted us to test whether other randomly selected mRNP proteins can also interact with mt tRNAs. Transiently expressed Flag-tagged SR proteins SRSF1, SRSF2, and SRSF3 and endogenous HeLa hnRNP A1 and H proteins were immunoprecipitated (Fig. 8A), and the associated RNAs were analyzed by gel electrophoresis (Fig. 8B). IP of each protein recovered a set of small RNAs falling into the size range of mt tRNAs. Northern blot analysis of the SR protein–associated RNAs with the complete set of mt tRNA oligonucleotide probes or hnRNP A1– and hnRNP H–associated RNAs with probes complementary to the five longest mt tRNAs demonstrated that the tested mRNP proteins interact with mt tRNAs (Fig. 8C,D). The hnRNP A1 and H bind predominantly to mt tRNAs Asn, Gln, and Phe. The SR proteins interact with many mt tRNAs, although SRSF1 associates most efficiently with mt tRNAs Val, Gln, and Asn, SRSF2 binds mainly to mt tRNA^Gln^, and SRSF3 interacts preferentially with mt tRNAs Phe, Val, Arg, and Ser (Agy). The identity of mt tRNAs shown in Figure 8B was inferred from their electrophoretic mobility and northern hybridization intensity. In transcriptionally arrested HeLa cells, both SR and hnRNP proteins showed increased associations with their mt tRNA partners, corroborating the conclusion that association of mRNP proteins with mt tRNAs is dynamic and it reversibly correlates with the transcriptional activity of the cell (Fig. 8E).

## DISCUSSION

Human cytoplasmic mRNAs dynamically associate with hundreds of RNA-binding proteins that control mRNA translation, stability, and localization (Singh et al. 2015). In this study, we have demonstrated that besides binding to cytoplasmic mRNAs, human mRNP proteins YBX1, YBX3, SRSF1, SRSF2, SRSF3, and hnRNP A1 and H also interact with mt tRNAs. Whereas YBX1, YBX3, and the three SR proteins bind to many mt tRNAs, hnRNP A1 and H interact predominantly with three mt tRNAs, Asn, Gln, and Phe (Figs. 1C, 8), and another earlier characterized mRNP protein, PTB, binds to mt tRNA^Thr^ with great specificity (Marnef et al. 2016). The mt tRNA-binding abilities of PTB, YBX1, and YBX3 were recognized accidentally. Because all other mRNP proteins selected for mt tRNA co-IP assays, SRSF1, SRSF2, SRSF3, and hnRNP A1 and H, have been found to interact with mt tRNAs (Fig. 8), we believe that many additional mRNP proteins possess mt tRNA binding capacity.

In vitro reconstitution and in vivo cross-linking experiments demonstrated that similar to PTB (Marnef et al. 2016), YBX1 and YBX3 directly interact with mt tRNAs (Figs. 3A,B, 4). All known mt tRNA-associated mRNP proteins possess RNA-binding capacity, and they carry one (SRSF2 and SRSF3), two (SRSF1, hnRNP A1 and H), or four (PTB) RNA recognition motifs (RRMs). The CSD of YBX1 and YBX3 also has RNA-binding affinity and encompasses the RNP1 and RNP2 consensus sequences present in RRMs (Sachs et al. 2012). Earlier, mutational analyses demonstrated that the RRM1 and RRM2 motifs of PTB recognize the D and T stem–loops of mt tRNA^Thr^ in an interdependent manner, and these two protein–RNA contacts provide high specificity for the PTB-mt tRNA^Thr^ interaction (Marnef et al. 2016). It is unclear whether both RRMs present in SRSF1, hnRNP A1, and H, participate in mt tRNA binding, but apparently, the single RRMs carried by SRSF2 and SRSF3 are able to support efficient mt tRNA binding. Mutations introduced into the RNA-binding surface of the CSD of YBX1 disrupt mt tRNA binding, demonstrating that the CSD coordinates mt tRNA binding (Fig. 4B,C). Similar to the RRM1 and RRM2 motifs of PTB, the CSD of YBX1 recognizes the T or D stem–loop regions of mt tRNAs (Fig. 4A). Compared to cyt tRNAs, mt tRNAs possess distinctive structural features, they fold into noncanonical cloverleaf structures that lack D loop/T loop interactions, or mt tRNA^Ser(AGY)^ lacks even the entire D stem–loop (Watanabe 2010; Suzuki et al. 2011). Thus, accessibility of the D and T stem–loops seems to be the major structural determinant of specific association of mt tRNAs with mRNP proteins.

Cell fractionation experiments demonstrated that YBX1, YBX3, and PTB interact with mt tRNAs in the cytosol outside of mitochondria (Fig. 5; Marnef et al. 2016). Most probably, SRSF1, SRSF2, SRSF3, and hnRNP A1 and H also interact with mt tRNAs in the cytoplasm, because none of these proteins has been detected in the mitochondria (Mootha et al. 2003; Pagliarini et al. 2008; Gaston et al. 2009). We have demonstrated that lysosomal degradation of superfluous or dysfunctional mitochondria, called mitophagy, represents at least one mechanism that can support mt tRNA release into the cytosol (Fig. 6). It remains unclear whether mt tRNAs are liberated from dysfunctional or damaged mitochondria before being targeted to autophagic degradation, or they can simply escape lysosomal RNA hydrolyses. About 30% of YBX1-associated mt tRNAs Phe and Lys lack an intact 3′-terminal CCA motif, suggesting that they undergo partial exonucleolytic degradation maybe during mitophagy or later in the cytosol. In this context, it is noteworthy that mt tRNAs transfected into HeLa cells show unexpectedly high metabolic stability that might be a consequence of their association with cytosolic mRNA proteins.

At the moment, the functional significance of cytosolic mt tRNA accumulation and association with mRNP proteins remains elusive. The observation that interaction of mt tRNAs and mRNP proteins is highly dynamic and it is governed by the actual transcriptional and physiological conditions of the cell point to a potential cytosolic regulatory role of mt tRNAs. It raises the fascinating possibility that mt tRNAs released into the cytosol in response to various physiological changes in the mitochondria may support a regulatory cross-talk between mitochondria and nuclear gene expression. Recently, cyt tRNA-derived fragments (tRFs) generated by the hypoxic stress–inducible endoribonuclease angiogenin have been proposed to control human mRNA translation and decay through binding to YBX1 (Fu et al. 2009; Thompson and Parker 2009; Yamasaki et al. 2009; Ivanov et al. 2011). In response to hypoxic stress, 5′ tRFs were proposed to destabilize some prooncogenic mRNAs by displacing their 3′ untranslated regions from YBX1 (Goodarzi et al. 2015). In principle, cytoplasmic mt tRNAs may also control mRNA stability, translation, and/or localization through competitive displacement of various mRNP proteins. It is also conceivable that mt tRNAs accumulating in the cytoplasm function simply as molecular decoys and may provide metabolic and/or conformational stability for mRNA-binding proteins dissociated from cytoplasmic mRNPs. Finally, YBX1 and other mRNP proteins may also bind to cytoplasmic mt tRNAs to target them to the exosomes and/or extracellular vesicles (Shurtleff et al. 2017).

Our results also support the possibility that mt tRNAs may have several different functions in the cytoplasm. During apoptosis, Cyt *c* and PTB show increased interactions with mt tRNAs in general and with mt tRNA^Thr^, respectively (Mei et al. 2010; Marnef et al. 2016). In contrast, YBX1 and YBX3 association with mt tRNAs is reduced in apoptotic cells (Fig. 7C). Although mt tRNAs Lys, Phe, and Leu(UUR) form only small, heterodimeric complexes with YBX1 and YBX3, the YBX1- and YBX3-mt tRNA^Val^ complexes are incorporated into larger (35S–60S) higher order structure(s) of unknown nature (Fig. 3D). Understanding of the cytoplasmic function(s) of mt tRNAs is a technically highly challenging task, because we cannot manipulate mt tRNA accumulation in the cytoplasm. Apparently, induction of mitophagy, although it augments mt tRNA concentrations in the cytoplasm, has complex physiological impacts on the cell. On the other hand, RNA interference or transfection of in vitro transcribed mt tRNAs are not efficient approaches to significantly modulate the interaction of HeLa mt tRNAs with YBX1 and other mRNP proteins (Marnef et al. 2016; B Jady, A Ketele, T Kiss, unpubl.). Dissection of the precise role of mt tRNA–mRNP protein complexes remains an important task for the future.

In summary, we have demonstrated that human mt tRNAs dynamically interact with cytoplasmic mRNP proteins. Although the functional role of cytoplasmic mt tRNA association with mRNP proteins remains unknown, our results provide strong support to the emerging view that besides mediating mitochondrial protein synthesis, human mt tRNAs released into the cytoplasm may possess additional important cellular functions.

## MATERIAL AND METHODS

### General procedures and cell cultures

Unless stated otherwise, standard laboratory protocols were used for manipulating RNA, DNA, oligonucleotides, and proteins. Oligodeoxynucleotides were purchased from Eurofins MWG. Mouse L929 and human HeLa, MCF7, MRC5V1, U2OS, VA13, and primary fibroblast cells were grown in Dulbecco's modified Eagle medium (DMEM) supplemented with 10% fetal calf serum (Invitrogen). RNA transcription was inhibited by incubation of HeLa cells with 20 µg/mL of α-amanitin (a-ama) (Sigma-Aldrich) for 3 h, with 100 µM of 5,6-dichloro-1-β-D-ribofuranosylbenzimidazole (DRB) (Sigma-Aldrich) and 1 µg/mL Actinomycin D (ActD) (Sigma-Aldrich) for 1 h. Apoptosis was induced by incubation of HeLa cells with 1 µM of STS (Sigma-Aldrich) for 4 h. To induce or to inhibit mitophagy, HeLa cells were incubated for 16 h with 1 µM of valinomycin or with 10 µM of chloroquine, respectively (Georgakopoulos et al. 2017). Transfection of HeLa cells with expression plasmids and in vitro transcribed RNAs was performed by using Lipofectamine 2000 (Invitrogen) as recommended by the manufacturer.

### Construction of expression plasmids

To obtain pFL-YBX1 and pXpress-YBX1, the full-length YBX1 cDNA was PCR-amplified and inserted into the HindIII and BamHI sites of the p3XFLAG-CMV (Sigma-Aldrich) or into the BamHI and XhoI sites of the pcDNA3.1/HisC (Invitrogen), respectively. To generate pHA-YBX1 and pHA-YBX3, YBX1 and YBX3 cDNAs were amplified using 5′-terminal primers encoding two copies of the influenza hemagglutinin (HA) tag peptide and the amplified DNAs were inserted into the *Hin*dIII and *Bam*HI sites of pcDNA3 (Invitrogen). The same approach was used for construction of pFL-YBX3 except that the 5′-terminal PCR primer encoded two copies of the Flag tag. To obtain pXpress-YBX1*CSDm*, appropriate point mutations were introduced into the pXpress-YBX1 expression plasmid by using PCR-mediated mutagenesis. To generate pFL-SRSF1, pFL-SRSF2, and pFL-SRSF3, full-length cDNAs of human SRSF1, SRSF2, and SRSF3 were inserted into the HindIII/EcoRI or EcoRI/BamHI (SRSF3) sites of p3XFLAG-CMV (Sigma-Aldrich).

### Immunoprecipitation, antibodies, and protein analyses

Preparation of cell extracts and IP of proteins were performed essentially as described before (Marnef et al. 2014, 2016). About 9 × 10^6^ cells were suspended in 0.5 mL of ice-cold NET-150 (50 mM Tris-HCl, pH 7.5, 150 mM NaCl, 0.05% Nonidet P-40) buffer and sonicated five times for 30 sec with 30-sec intervals with a Bioruptor Plus sonicator (Diagenode) at high setting. Cell debris were removed by centrifugation at 16,000*g* for 10 min, and in some experiments, the extracts were further clarified from large RNPs by centrifugation at 100,000*g* for 20 min. Cytosolic extract was prepared by mild digitonin extraction of HeLa cells. About 9 × 10^6^ PBS-washed cells were resuspended in 500 µL of digitonin extraction buffer (50 mM HEPES, pH 7.4, 150 mM NaCl, 1 M hexylene glycol, 25 µg/mL digitonin) and gently agitated by end-over-end rotation for 15 min at 4°C. Cells were removed by centrifugation at 2000*g* for 10 min at 4°C and the supernatant was further clarified by centrifugation at 13,000*g* for 10 min at 4°C. For IP, 20 µL of packed protein A Agarose beads (Sigma-Aldrich) coupled with 5 µL of monoclonal anti-YBX1 (ab76149, Abcam), anti-mYBX1 (ab12148, Abcam), anti-hnRNP A1 (sc32301, Santa Cruz), anti-hnRNP H (ab10374, Abcam), anti-Flag (F3165, Sigma-Aldrich) and anti-HA (clone HA-7, Sigma-Aldrich) and polyclonal anti-YBX3 antibodies (produced by Agro-Bio) were incubated with 450 µL cell extract for 1 h at 4°C. Beads were washed four times with ice-cold NET-150. In vivo RNA–protein cross-linking was performed as described before (Niranjanakumari et al. 2002), except that the RNA IP (RIPA) and wash buffers contained 0.2% sodium dodecyl sulfate (SDS). For western blot analyses, the following antibodies were used in the indicated dilutions: monoclonal anti-YBX1 (1:4,000) (ab12148, Abcam), polyclonal anti-YBX3 (1:2,000) (Agro-Bio), monoclonal anti-Flag-HRP (1:100,000) (A8592, Sigma-Aldrich), monoclonal anti-HA-HRP (1:5,000) (ABIN 1573874, GenScript), monoclonal anti-Xpress (1:5,000) (Invitrogen), monoclonal anti-hnRNPA1 (1:2,000) (sc32301, Santa Cruz), monoclonal anti-hnRNP H (1:3,000) (ab10374, Abcam), anti-ATP5A1 (1:1000) (459240, Invitrogen), anti-TOM20 (1:1000) (612278, BD Transduction Laboratories), anti-Cyt C (556433, BD Pharmingen) (1:500).

### RNA analyses

From cell extracts and IP pellets, RNAs were isolated by phenol–chloroform extraction. For northern blot analyses, RNAs were fractionated on 6% sequencing gels, electroblotted onto a Hybond-N nylon membrane (GE Healthcare) and probed with 5′ end-labeled sequence-specific oligodeoxynucleotides (sequences are available upon request). For slot blot analyses, RNAs recovered from the pellets of YBX1 and YBX3 IP reactions were immobilized on a nylon membrane using a slot blot apparatus and probed with radiolabeled oligonucleotides. The hybridization reactions were analyzed by PhosphorImager quantitation. In vitro 3′ end labeling of RNAs with [5′-^32^P]pCp and T4 RNA ligase (Thermo Scientific) and chemical sequencing of terminally labeled, gel-purified mt tRNAs have been described (Kiss et al. 1985; Kiss and Solymosy 1990). RNA 3′ end race analysis has been reported (Kiss and Filipowicz 1993).

### In vitro transcription of wild-type and mutant mt tRNAs

Template DNAs for in vitro synthesis of internally labeled wild-type and mutant mt tRNAs and cyt tRNA^Gln(TTG)^ were generated by PCR amplification with appropriately designed oligodeoxynucleotide primers incorporating the bacteriophage T7 promoter. In vitro RNA synthesis was performed in the presence of 0.2 µg of template DNA, 10 units of T7 RNA polymerase (Promega), and 50 µCi of [α-^32^P]-CTP (specific activity 30–40 Ci/mmol). For synthesis of cold RNAs, [α-^32^P]-CTP was omitted. Before utilization, each RNA product was purified on a 6% sequencing gel.

### Recombinant YBX1 and YBX3 purification and electrophoretic mobility shift assay

To generate pSCodon-YBX1 and pSCodon-YBX3 bacterial expression vectors, YBX1 and YBX3 cDNAs were amplified by RT-PCR using 5′ primers encoding the Strep-Tactin tag and the resulting DNA fragments were inserted into the BamH1 and HindIII sites of pSCodon1 (Delphi Genetics). A similar approach was used to produce pSCodon-YBX1*CSDm,* except that the pXpress-YBX1*CSDm* expression plasmid was used as a template for PCR amplification. *E. coli* Rosetta strains transformed with the pSCodon-YBX1, pSCodon-YBX3, or pSCodon-YBX1*CSDm* expression plasmids were grown overnight at 18°C in 5 L of 2× YT medium complemented with 100 µg/mL ampicillin, 100 µg/mL chloramphenicol, and 0.3 mM IPTG. Cells were harvested and resuspended in 200 mL of 50 mM NaH_2_PO_4_, 300 mM NaCl, 1 mM imidazole (pH 8.0) solution, and they were disrupted by freezing and sonication four times for 30 sec by a Branson sonifier at setting 5. After centrifugation at 9000*g* for 25 min, the clarified extracts were incubated with 1 mL of Ni-NTA-agarose beads (Qiagen) for 1 h at 4°C. Beads were washed four times with 50 mM NaH_2_PO_4_, 300 mM NaCl, and 10 mM imidazole (pH 8.0) buffer and the bound proteins were eluted with 50 mM NaH_2_PO_4_, 300 mM NaCl, and 150 mM imidazole (pH 8.0). Proteins were loaded on a Strep-Tactin Superflow Plus cartridge (Qiagen) and were washed twice with 50 mM NaH_2_PO_4,_ pH 8.0, containing first 300 mM then 150 mM NaCl. Proteins were eluted with 50 mM NaH_2_PO_4_, 150 mM NaCl (pH 8.0) solution complemented with 2.5 mM desthiobiotin. The purified proteins were frozen and kept at −80°C in 20% glycerol. Electrophoretic mobility shift assay has been described before (Marnef et al. 2016).

### Cell fractionation

HeLa cells (∼2 × 10^7^) were suspended in 2 mL of ice-cold hypotonic buffer A (10 mM HEPES-KOH, pH 7.9, 1.5 mM MgCl_2_, 10 mM KCl, 0.5 mM dithiothreitol) and incubated on ice for 10 min. Cells were broken in a Dounce homogenizer using pestle B by 15 strokes and the homogenate was centrifuged at 600*g* for 5 min. The obtained pellet and supernatant were considered as crude nuclear and cytoplasmic fractions, respectively. Nuclei were washed once in nuclei wash buffer (10 mM Tris-HCl, pH 7.5, 3.3 mM MgCl_2_, 250 mM sucrose), resuspended in 0.5 mL of NET-150, and sonicated five times for 30 sec with 30-sec intervals by a Bioruptor Plus sonicator (Diagenode) at high setting. The resulting homogenate was clarified by centrifugation at 16,000*g* for 10 min to generate a soluble nuclear extract. The crude cytoplasmic fraction was further clarified by three subsequent centrifugation steps at 1000*g*, 10,000*g*, and 16,000*g* for 5 min in each case. Finally, the obtained soluble cytoplasmic extract was supplemented with 40 µL of 5 M NaCl and 0.5 µL of Nonidet P-40.

For preparation of mitochondria, HeLa cells (∼2 × 10^7^) were washed in TD buffer (25 mM Tris-HCl, pH 7.5, 133 mM NaCl, 5 mM KCl, 0.7 mM Na_2_HPO_4_,) and resuspended in 2 mL of RSB buffer (10 mM Tris-HCl, pH 7.5, 10 mM NaCl, 1.5 mM CaCl_2_). Cells were incubated on ice for 10 min and homogenized with a Dounce homogenizer using pestle B by 15 strokes. The extract was completed with 1.3 mL, 2.5× MS buffer (12.5 mM Tris-HCl, pH 7.5, 525 mM mannitol, 175 mM sucrose, 12.5 mM EDTA). Nuclei were removed by three sequential centrifugations at 1250*g* for 5 min at 4°C and mitochondria were collected by centrifugation at 20,000*g*, for 15 min at 4°C. The mitochondrial pellet was resuspended in 1 mL of 50 mM Tris-HCl, pH 7.5, 10 mM EDTA and 20% sucrose, and layered onto a sucrose cushion composed of two layers of 7 mL 1.5 M and 1.0 M sucrose solutions in 10 mM Tris-HCl, 5 mM EDTA. After centrifugation at 65,000*g* for 20 min, mitochondria were recovered from the interphase of the two sucrose cushions with a Pasteur pipette, supplemented with 0.5 mL 1× MS buffer, and collected by centrifugation at 20,000*g* for 10 min at 4°C.

### Sucrose gradient analysis

HeLa cells (∼2 × 10^7^) were suspended in 500 µL of NET-150 buffer and sonicated five times for 30 sec with 30-sec intervals in a Bioruptor Plus sonicator (Diagenode) at high setting. The cell homogenates were clarified by centrifugation at 16,000*g*, for 10 min at 4°C. Extracts corresponding to 2 mg of cellular proteins were layered on top of a 10%–50% sucrose gradient containing 10 mM Tris-HCl, pH 7.5, 150 mM NaCl, and 1.5 mM MgCl_2_ and centrifuged in a SW41 Ti swing-out rotor at 188,000*g* for 150 min. The gradients were fractionated into 14 fractions by an ISCO UA-6 gradient collector.

### Fluorescent in situ hybridization microscopy

Oligodeoxynucleotides A*GTGTATTGCTTTGAGGAGGTAAGCTACAT* (Gln), A*ATGGGGTGATGTGAGCCCGTCTAAACATA* (Phe1) and A*AGTGTATTGCTTTGAGGAGGTAAGCTACATA* (Phe2) carrying C6-aminoallyl-modified nucleotides (marked by asterisks) were labeled with FluoroLink Cy3 monofunctional reactive dye (GE Healthcare) and were used as probes for in situ hybridization to mitochondrial tRNAs (http://www.einstein.yu.edu/labs/robert-singer/protocols/). Hybridization was carried out in 2× SSC containing 20% formamide. Mitochondria were detected with anti-ATP5A1 (1:100 dilution) (459240, Invitrogen). Slides were mounted in mounting media (90% glycerol, 1× PBS, 0.1 µg/mL DAPI, 1 mg/mL *p*-phenylenediamin). Raw images were acquired using NIS-Elements AR software on a Nikon TI-E/B inverse microscope with CFI Plan APO VC 100× 1.4 objective and a Hamamatsu OrcaR2 CCD camera (binning 1 or 2). Final images were prepared with Adobe Photoshop.

## SUPPLEMENTAL MATERIAL

Supplemental material is available for this article.

## Supplementary Material

Supplemental Material
