# Supplementary material for: Dynamic association of human mRNP proteins with mitochondrial tRNAs in the cytosol
Source: RNA. 2018 Dec;24(12):1706–20. doi: 10.1261/rna.066738.118 (PMC6239184; doi:10.1261/rna.066738.118)
Supplement: Supplemental Material [file supp_24_12_1706__index.html]

Dynamic association of human mRNP proteins with mitochondrial tRNAs in the cytosol — Supplemental Material 

# Dynamic association of human mRNP proteins with mitochondrial tRNAs in the cytosol

## Supplemental Material

- Supplemental\_Material\_.pdf
