# Supplementary material for: Dynamic association of human mRNP proteins with mitochondrial tRNAs in the cytosol
Source: RNA. 2018 Dec;24(12):1706–20. doi: 10.1261/rna.066738.118 (PMC6239184; doi:10.1261/rna.066738.118)
Supplement: Supplemental Material [file supp_066738.118_Supplemental_Material_.pdf]

Supplemental Material

Dynamic association of human mRNP proteins with mitochondrial tRNAs in the cytosol

Beáta E. Jády[1,] Amandine Ketele[1] and Tamás Kiss[1,2,*]

[1]Laboratoire de Biologie Moléculaire Eucaryote du CNRS, Centre de Biologie Intégrative, Université de Toulouse, CNRS, UPS, 118 route de Narbonne, 31062 Toulouse Cedex 9, France, [2]Biological Research Centre, Hungarian Academy of Sciences, Szeged, Hungary

[*] To whom correspondence may be addressed:

e-mail: tamas@ibcg.biotoul.fr

Phone: (33) 56 133 59 07

Fax: (33) 56 133 58 86

Supplemental Figure S1

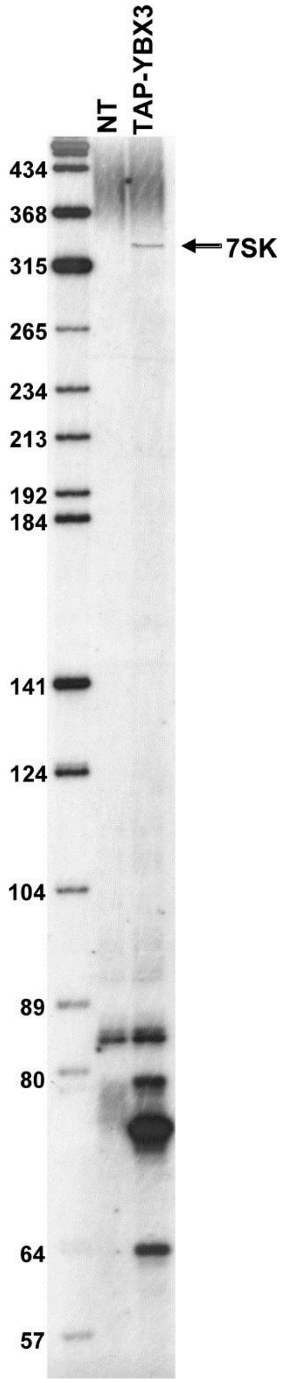

Supplemental Fig. S1. HeLa small RNAs interacting with transiently expressed TAP-YBX3. Cellular extracts prepared from HeLa cells non-transfected (NT) or transfected with the pTAP-YBX3 expression plasmid expressing TAP- (tandem affinity purification)-tagged YBX3 were incubated with IgG Sepharose (IP). After centrifugation, RNAs co-purified with TAP-YBX3 were 3' end-labeled with [5'-^{32}P]pCp and T4 RNA ligase and size-fractionated on a 6% sequencing gel. The pTAP-YBX3 expression construct was kindly provided by Dr. K. Collins (Hogg and Collins 2007). The 7SK snRNA is indicated. Lane M, size markers in nucleotides.

Supplemental Figure S2

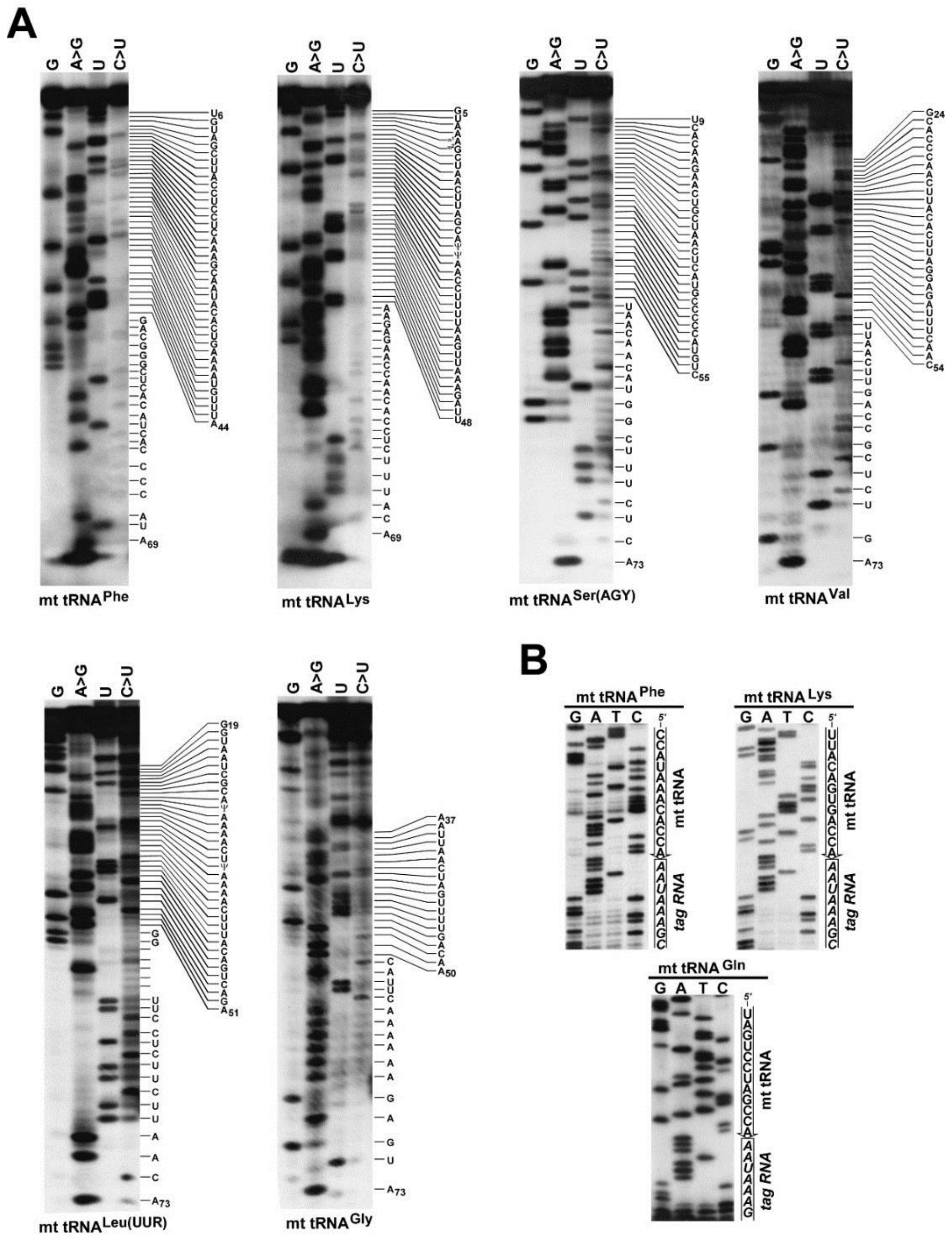

Supplemental Fig. S2. Identification of YBX1-associated RNAs. **A:** RNA sequence analysis. HeLa YBX1-associated 3' end-labeled small RNAs were separated on a 6% sequencing gel. After excision and elution from the gel, RNAs were subjected to base-specific partial chemical degradation as indicated above the lanes. The RNA degradation products were separated on 8% sequencing gels. The nucleotide sequences of human mt tRNAs are indicated and numbered according to (Sprinzl et al. 1998). Reported modifications are indicated. Perfect copies of the obtained mt tRNA sequences are in the human mitochondrial genome, excluding the formal possibility that these RNAs are transcribed from mitochondrial DNAs integrated into the human nuclear genome which carry nucleotide alterations. **B:** Determination of the 3'-terminal sequences of human YBX1-associated mt tRNAs Phe, Lys and Gln by 3' end race. The tag RNA and mt tRNA sequences are indicated.

Supplemental Figure S3

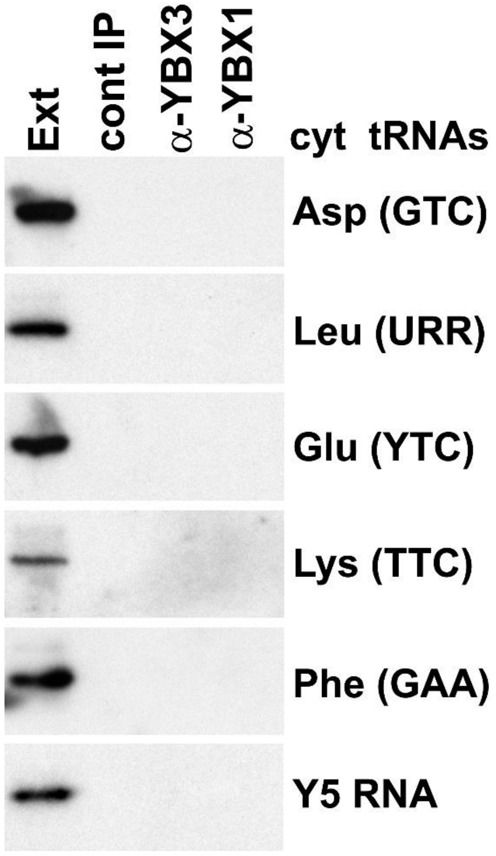

Supplemental Fig. S3. YBX1 and XBX3 do not associate with cyt tRNAs. RNAs co-immunoprecipitated with YBX1 and YBX3 from a HeLa cell extract (Ext) were separated on a 6% sequencing gel and electroblotted onto a nylon membrane (see Fig. 1 C). The membrane were probed with terminally labelled oligonucleotide probes specific for cyt tRNAs Asp(GTC), Leu(URR), Glu(YTC), Lys (TTC) and Phe (GAA) as well as for the Y5 small cytoplasmic RNA. Lane cont IP, immunoprecipitation with non-immune sera.

Supplemental Figure S4

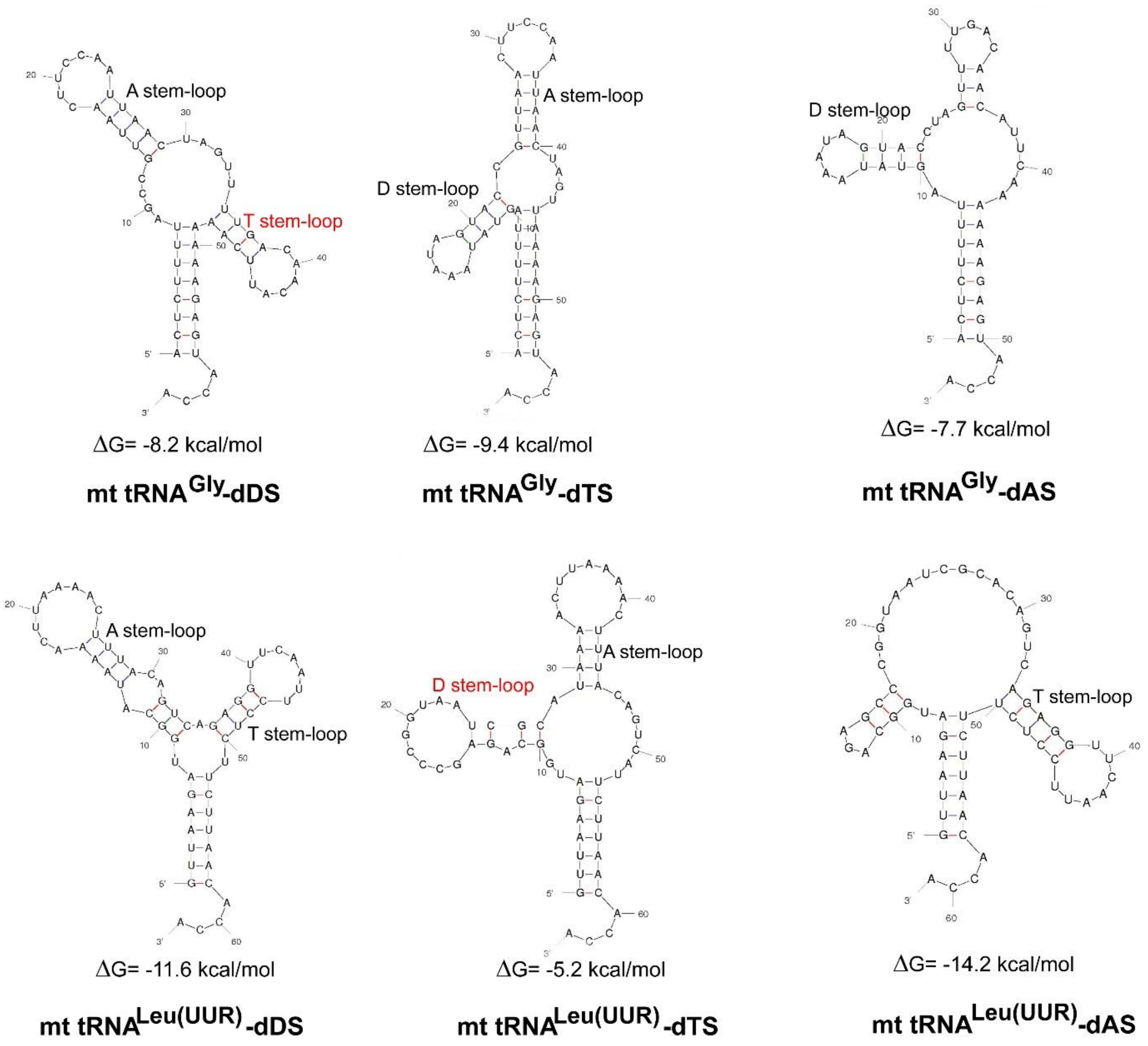

Supplemental Fig. S4. Predicted secondary structures of *in vitro* synthesized, internally truncated human mt tRNAs used for transfection of HeLa cells.

Supplemental Figure S5

RNA	Min	Max
mt tRNA Phe	0.001550	0.002444
mt tRNA Lys	0.001504	0.004190
mt tRNA Leu	0.001244	0.002409
mt tRNA Gly	0.006120	0.011913
mt tRNA Val	0.000770	0.007446

Supplemental Fig. S5. Relative cytoplasmic accumulation of selected mt tRNAs. The relative concentrations of mt tRNAs Phe, Lys, Leu(UUR), Gly and Val in cytoplasmic digitonin extracts were measured by slot blot analyses in eight independent experiments. After correction with the extraction efficiency of cyt tRNAs Gln and Thr, the obtained values were compared to total mt tRNA levels measured in cell sonic extracts. The ratios of the cytosolic mt tRNA fractions relative to the total amounts of mt tRNAs were calculated by the fitting linear models in RStudio (v0.99.903 with R v3.3.1) and were estimated with 95% confidence intervals.

Supplemental Figure S6

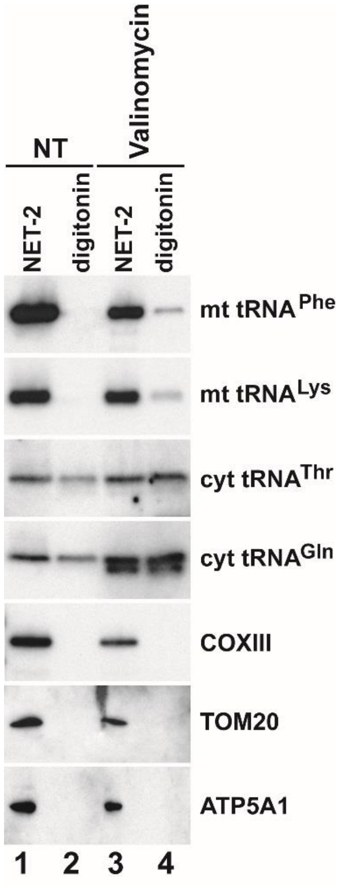

Supplemental Fig. S6. A digitonin extract prepared from valinomycin-treated cells lacks COXIII mitochondrial mRNA, TOM20 and ATP5A1 mitochondrial proteins. From human HeLa cells either non-treated (NT) or treated with valinomycin, total sonic (NET-2) and digitonin extracts were prepared. Distributions of mt tRNAs Phe and Lys, cyt tRNAs Thr and Gln (northern blot analysis), mitochondrial COXIII mRNA (RNase A/T1 mapping) and TOM20 and ATP5A1 proteins (western blot analysis) were compared.

Supplemental Figure S7

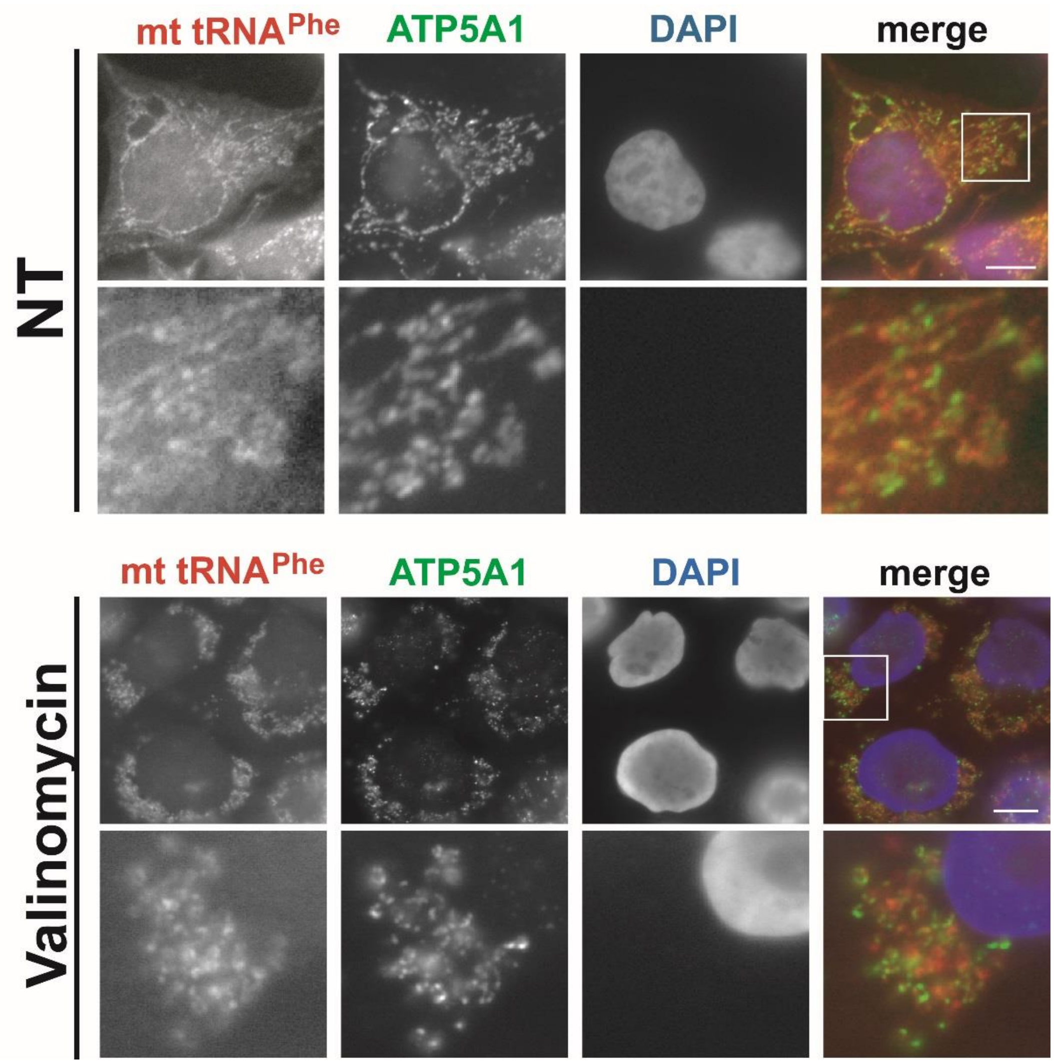

Supplemental Fig. S7. Cytoplasmic distribution of mt tRNA^{Phe} after induction of mitophagy. HeLa cells were either treated or non-treated (NT) with valinomycin before hybridization with mixture of two fluorescently labeled oligodeoxynucleotides complementary to mt tRNA^{Phe}. Mitochondria were stained with antibodies against ATP5A1. Bars represent 10 µm. Lower panels show enhanced magnifications of the boxed cytoplasmic areas (13,4 x 13,4 µm). Nuclei were visualized with DAPI staining.

Supplemental references

Hogg JR, Collins K. 2007. RNA-based affinity purification reveals 7SK RNPs with distinct composition and regulation. *RNA* **13**: 868-880.

Sprinzl M, Horn C, Brown M, Ioudovitch A, Steinberg S. 1998. Compilation of tRNA sequences and sequences of tRNA genes. *Nucleic Acids Res* **26**: 148-153.
